# Waste to Alternative Fuel: Experimental Investigation of the Effects of Pyrolytic Oil Obtained from Waste Tires, Waste Transformer Oil, and Diesel Fuel Ternary Blends on Engine Performance and Exhaust Emissions in a Diesel Engine

**DOI:** 10.1021/acsomega.6c01115

**Published:** 2026-05-22

**Authors:** Muhammet Kaan Yesilyurt, İlhan Volkan Öner, Tuba Hatice Doğan, Orhan Arpa, Mustafa Köksal İnce

**Affiliations:** † Department of Mechanical Engineering, Faculty of Engineering, Atatürk University, Erzurum 25240, Turkiye; ‡ Department of Mechanical Engineering, Faculty of Engineering, Adana Alparslan Türkeş Science and Technology University, Adana 01250, Turkiye; § Volkan Engineering, Ata Teknokent, Erzurum 25240, Turkiye; ∥ Department of Chemical Engineering, Faculty of Engineering, Atatürk University, Erzurum 25240, Turkiye; ⊥ Department of Mechanical Engineering, Faculty of Engineering, Dicle University, Diyarbakır 21280, Turkiye; # Graduate School of Natural and Applied Sciences, Atatürk University, Erzurum 25240, Turkiye

## Abstract

In recent years, the worsened environmental impacts and the limited reserve of fossil fuels have increased interest in alternative fuels. Fuels obtained by recycling waste streams, such as waste tires and transformer oils, are promising options. Pyrolytic oil from end-of-life waste tires is not an alternative for diesel engines on its own, but its fuel blends with diesel in different proportions enable its use in diesel engines without any modification. Furthermore, transformer oils used as heat-transfer fluids in electrical transformers reach the end of their useful life after completing a certain number of cycles and become waste. This waste stream can also be used to blend diesel fuel. In this study, waste transformer oil (WTRO) and pyrolytic oil from waste tires (WTPO) were mixed with diesel fuel in different ratios to prepare binary and ternary blends: WTRO30, WTRO10WTPO20, WTRO20WTPO10, and WTPO30. The performance and emissions of these fuel blends were investigated on a diesel engine. To avoid knocking, a cetane-number improver was added to all blends at a volume ratio of 1%. In addition, detailed analyses of certain physicochemical and thermochemical properties (FT-IR, density, kinematic viscosity, cloud point, pour point, and lower heating value) of all blends were performed. The results of the study showed that the chemical properties of the prepared blends exhibited similar results to diesel fuel. When compared with pure diesel data, the most significant performance loss across all speed ranges was observed in the WTPO30 fuel sample. The use of WTPO30 resulted in a noticeable decrease in maximum torque and a brake power while recording the highest brake specific fuel consumption and lowest brake thermal efficiency. Furthermore, significant increases in CO, NO, and NO_*x*_ emissions were observed. The results of the study revealed striking findings regarding the use of WTPO and WTRO in diesel engines.

## Graphical abstract

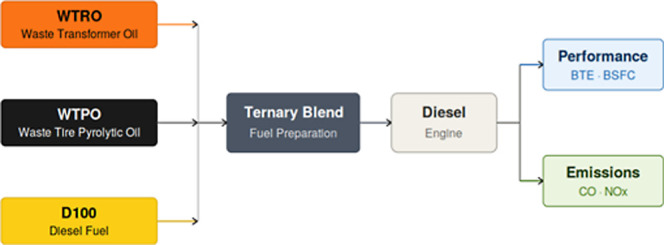

## 1 Introduction

The rapid depletion of fossil fuel reserves and increasingly stringent environmental regulations have accelerated the search for sustainable alternative fuels for internal combustion engines. In this context, waste tire pyrolytic oil (WTPO) obtained from end-of-life tires and waste transformer oil (WTRO) from electrical transformers offer enormous potential in terms of waste management and energy recovery. Despite their high energy content, both WTPO and WTRO are not suitable for direct (unmodified) 100% use in diesel engines due to physicochemical drawbacks such as a low cetane number and high kinematic viscosity, respectively. Therefore, blending both types of waste oil with conventional diesel fuel or other alternative fuels in specific ratios stands out as the most rational solution for waste disposal without requiring any hardware modifications to engine systems.

Early WTRO studies in the literature focused on understanding the key effects of directly adding this oil to diesel fuel on the engine performance. Belkhode et al.^1^ reported that BTE gradually decreased and BSFC values increased as the WTRO ratio increased in WTRO-diesel blends processed by atmospheric distillation. However, subjecting WTRO to appropriate thermochemical processes largely eliminates these disadvantages. For example, Nabi et al.^2^ have proven that WTRO, which is subjected to the transesterification process, has basic properties such as density, viscosity, and cetane number very close to those of diesel fuel and exhibits a performance equivalent to that of reference diesel in engine tests. Similarly, Behera and Murugan^3^ confirmed that by optimizing WTRO-diesel blends prepared at different volumetric ratios, an increase in BTE and a reduction in smoke emissions can be achieved compared to pure diesel.

To further improve the combustion dynamics of the WTRO, researchers have focused on fuel processing techniques as well as hardware and operational modifications. Yadav et al.^4^ demonstrated that combustion chamber geometry plays a critical role in the use of catalytic cracking recovered WTRO (at a rate of 50%); it found that using recirculating combustion chambers (RCC) instead of conventional hemispherical open combustion chambers (HCC) increased BTE by 3% and resulted in reductions of 8%, 6%, and 5% in HC, CO, and smoke emissions, respectively. On the operational optimization side, Yadav et al.^5^ reported that delaying the injection timing in an engine running with transesterified WTRO resulted in significant reductions in NO_*x*_, CO, and unburned hydrocarbon emissions but increased smoke emissions. In addition, dual-fuel strategies have been tried to compensate for the low reactivity of WTRO; Behera et al.,^6^ in their studies using WTRO as a pilot fuel and adding acetylene to the intake air, proved that the ignition delay time was shortened, and BTE was increased under full load conditions, reducing smoke formation.

Nanotechnology and hydrogen enrichment strategies have come to the fore in recent years to increase the reactivity and combustion efficiency of the WTRO. Karthikeyan et al.^7^ achieved a 10.4% increase in BTE compared to pure diesel when they added hydrogen (6 L/min) together with TiO_2_ and SiO_2_ nanoparticles to WTRO–diesel mixtures; they recorded reductions of up to 40% in CO, unburned hydrocarbon (UHC), and smoke emissions. However, they also noted that these improvements led to a 28% increase in the level of NO_*x*_. Similarly, Sathish et al.^8^ added TiO_2_ to the mixture containing 50% WTRO and supported it with hydrogen; they reported a massive increase of 37.30% in BTE and a decrease of 15.33% in BSFC. The integration of exhaust gas recirculation (EGR) systems has also been tested to control increasing NO_*x*_ emissions. Sathish et al.^9^ added ZnO and CeO_2_ nanoparticles to WTRO fuel and applied 20% EGR, showing that the nanoparticles reduced CO and smoke emissions and EGR successfully suppressed NO_*x*_ formation.

The approach of solving the disadvantages of WTRO by blending it with other waste-derived biodiesels with high oxygen content instead of complex nanoadditives is also widely discussed in the literature. Sethuraman et al.^10^ mixed WTRO and waste frying oil methyl esters with diesel; the study found that the B20 blend increased BTE while decreasing BSFC, CO, and HC emissions but increased NO_*x*_ emissions. Büyükoğlu et al.^11^ focused on multicomponent blends and confirmed that by blending waste olive oil biodiesel with WTRO, these blends resulted in significant reductions in NO_*x*_ emissions, although they created a slight disadvantage compared to diesel in terms of power and torque. In addition, Şen et al.^12^ examined the direct effect of the WTRO ratio and reported that low WTRO ratio (TD10) mixtures reduced BSFC by 6.32% and increased BP by 9.9%; however, when the ratio increased to 40% (TD40), the torque decreased by 14.03%, thus emphasizing the vital importance of the “optimum mixture ratio”.

On the other hand, waste tire pyrolytic oil (WTPO) obtained from waste tires is often blended with diesel or biodiesel due to its poor combustion properties despite its high energy density, and predictive modeling is increasingly used to find the optimum blend in this process. Mickevicius et al.^13^ mixed WTPO with hydrogenated vegetable oil (HVO) and achieved increases in BTE of up to 30.8%, modeling these results with high accuracy using machine learning (ML) algorithms such as linear regression and random forest. Using a similar optimization approach, Sahin et al.^14^ investigated low-ratio WTPO-diesel blends using both ML and response surface methodology (RSM); studies have shown that the addition of WTPO increases BSFC and NO_*x*_ emissions, but that CO_2_ can be minimized at appropriate engine speeds. Using RSM to solve the complexity of quaternary mixtures, Chow et al.^15^ optimized a combination of diesel, palm oil biodiesel, diethyl ether (DEE), and WTPO; the study found that a mixture containing 5% WTPO and 2.091% DEE reduced BSFC by 14% and CO by 22.7%.

The main limitations on the direct use of WTPO in commercial diesel engines are described by Mikulski et al.^16^ It has been clearly demonstrated that high sulfur and polycyclic aromatic hydrocarbon content increases particulate emissions. Nevertheless, numerous studies have confirmed that low-ratio blends can be used as a drop-in fuel without the need for engine modification. For example, Hürdoğan et al.^17^ reported that WTPO–diesel blends in a four-cylinder naturally aspirated engine exhibited performance quite close to pure diesel in terms of torque and power. Similarly, in their tests with commercial diesels with different cetane numbers (CN48, CN51, and CN53), Khairil et al.^18^ found that adding up to 20% WTPO was the optimum limit to maintain engine performance. Furthermore, Nabi et al.^19^ proved the predictability of these mixtures by reporting that the thermophysical properties of multicomponent mixtures containing WTPO converge to diesel fuel and that there is a maximum deviation of 10% between experimental results and numerical modeling.

Although low-ratio blends maintain overall performance, the low cetane number of WTPO has specific and challenging effects on combustion dynamics. Uyumaz et al.^20^ tested the W10 mixture they obtained by optimizing the pyrolysis parameters in a direct injection engine; they observed that the ignition delay time, which shortens depending on the load in diesel, is extended with the addition of WTPO, and the maximum BTE is reduced from 28.27% to 25.12%. This delayed ignition phenomenon leads to sudden increases in cylinder pressure and heat release rates. Indeed, Karagöz et al.^21^ has investigated in detail how WTPO ratios ranging from 10% to 50% affect not only the performance (increased BSFC and decreased BTE) and emissions but also the NVH (noise, vibration, and harshness) characteristics of the engine. The study revealed that extending the ignition delay period with the addition of WTPO increased the peak HRR_max_ and CPmax values; this sudden and intense combustion directly increased the vibration and noise levels in the engine. The same study observed a slight increase in NO_*x*_ emissions, while HC emissions were reported to have gradually decreased.

To mitigate the delayed combustion, high emissions, and NVH problems caused by WTPO, ternary blends, which incorporate alcohols with high oxygen content (especially butanol), have gained significant importance. Karagöz^22^ added high proportions of *n*-butanol to WTPO–diesel blends and showed that n-butanol successfully suppressed the increases in NO_*x*_, CO, and HC caused by WTPO thanks to its suitable density, viscosity, and oxygen content. Furthermore, this alcohol addition contributed to an increase in BTE while reducing BSFC; however, the lowest CO emissions were still measured in pure diesel under all load conditions. Öner et al.^23^ applied a similar triple blending strategy using crude pyrolytic oil (CPO) and tested diesel, butanol, and CPO mixtures at different engine loads. The results showed that the engine could operate stably without knocking even when the CPO ratio reached 50%; the highest BSFC (425.35 g/kWh) was recorded in the PO30Bu20CN1 sample; and a reasonable average reduction of 8.28% was observed in BTE compared to diesel.

In addition to alcohols, blending other waste-derived biodiesels or alternative fuels with WTPO is also an effective method for balancing performance. Ağbulut et al.^24^ found that WTPO–diesel blends caused a 9.13% decrease in BTE, a 21.78% increase in BSFC, and a 7.09% increase in CO and NO emissions, respectively. However, when waste frying oil methyl ester (biodiesel) was added to this mixture, BTE recovered by 7.51%, BSFC increase was offset by 8.89%, CO emissions decreased by 7.69%, and NO increase was limited to 4.64%. The same study reported that adding waste rocket fuel also provided similar improvements. Karthikeyan et al.^25^ blended WTPO with a different biodiesel source (*Codium decorticatum*) and reported that biodiesel–WTPO blends in certain ratios minimize ignition delay time and low oxygen content reduces NO_*x*_ emissions. Although CO and CO_2_ emissions remain at low levels, the necessity of exhaust gas control systems for the stable operation of the system has been emphasized.

In addition to alcohol and biodiesel blends, gas-assisted dual-fuel systems have also been investigated to compensate for WTPO’s low calorific value and poor combustion characteristics. Wu et al.^26^ injected hydrogen into WTPO-biodiesel blends from the intake manifold and showed that hydrogen successfully balanced the effective energy content of low calorific value fuels. Similarly, Hoang et al.^27^ tested mixtures containing 50% biodiesel and 50% pyrolysis oil in a homogeneous charge compression ignition (HCCI) engine and continuously fed the system with compressed natural gas (CNG) (3 L/min). Experimental results have shown that, despite this complex strategy, BTE lags behind pure diesel, highlighting the difficulty of controlling the combustion phase of WTPO.

One of the most popular and effective methods to overcome these challenges in recent times is the addition of metal-oxide nanoparticles as fuel additives (nanoadditives). Additives for improving the fuel properties of WTPO were discussed by Ayanoğlu and Yumrutaş.^28^ Distillation properties of blends made with natural zeolite and lime additives were examined, and it was determined that the sample containing 10% lime exhibited the closest properties to diesel fuel. It was reported that 18% of the products obtained as a result of distillation consisted of gasoline-like, 70% of diesel-like, and 12% of residual components.^29^

Polat^29^ succeeded in increasing BTE by adding Al_2_O_3_ nanoparticles to WTPO–diesel mixtures while achieving simultaneous reductions in in-cylinder maximum pressure, heat release rate (HRR), BSFC, exhaust temperature, and HC, CO, NO_*x*_ emissions. Similar improvements were made by Sathiyamoorthi et al.^30^ in mixtures of alumina nanoparticles with B20, and this was confirmed by Prajapati et al.^31^ with SrO nanoparticles added to waste frying oil biodiesel. Also, Kumar et al.^32^ demonstrated the microexplosion and catalytic effects of nanoadditives by reducing BSFC by 12% and reporting 16.3% less NO_*x*_ emissions compared to diesel by integrating 1-decanol and TiO_2_ nanoparticles into WTPO.

As can be seen, the use of waste-derived oils such as WTRO and WTPO in diesel engines is technically possible; however, performance and emission characteristics are strongly dependent on the fuel treatment method applied, the blending ratio, and the costly additives used. When the literature is examined, WTRO is generally used in binary blends with biodiesel, butanol, or hydrogen;^4,7,8^ WTPO is also studied in binary or multiple forms with diesel, butanol, or various nanoparticles.^15,23,26^ However, studies in the literature that simultaneously and homogeneously mix both industrial waste oils (WTRO and WTPO) with diesel fuel to form a ternary blend, and subject their synergistic effects on engine performance/exhaust emissions to detailed experimental analyses, are quite limited. Current research reveals a significant gap in understanding how these two waste oils can balance each other’s physicochemical drawbacks (e.g., the high viscosity of WTRO and the aromatic nature of WTPO).

This study aims to fill this critical gap in the literature. Unlike previous studies, in this work, five different test samples (D100, WTRO30, WTRO10WTPO20, WTRO20WTPO10, and WTPO30) were prepared by homogenizing WTRO and WTPO with diesel fuel in specific volumetric ratios without the need for complex and costly nanoadditives. The main objective of this study is to analyze the chemical characteristics (FT-IR, distillation curve, etc.) of these unique ternary mixtures and to comprehensively compare their performance and exhaust emission profiles in a single-cylinder diesel engine. The aim is to demonstrate the potential of triple blends with balanced ratios (e.g., WTRO10WTPO20) to maximize body energy transfer and minimize specific emissions in an environmentally friendly way by compensating for the negative characteristics of waste oils.

## 2 Materials and Methods

### 2.1 Fuel Blends and Characterization

WTRO and WTPO used in the experiments were supplied by TEİAŞ Erzurum Regional Directorate and Era Environmental Technologies Inc., respectively. Before preparing the blends, these samples were filtered to ensure fuel quality, removing any sediment and water content. As shown in Table 1, five different blends were prepared.

**Table 1:** Volumetric Ratios and Quantities of the Prepared Blends^a^

sample no.	fuel blend	WTPO (%v/v)	WTRO (%v/v)	diesel (%v/v)	WTPO (mL)	WTRO (mL)	diesel (mL)	total (mL)
1	D100	0	0	100	0	0	2500	2500
2	WTRO30	0	30	70	0	750	1750	2500
3	WTRO10WTPO20	20	10	70	500	250	1750	2500
4	WTRO20WTPO10	10	20	70	250	500	1750	2500
5	WTPO30	30	0	70	750	0	1750	2500

a For use in experimental studies, 2500 mL samples were prepared from each blend. To achieve complete homogeneity without phase separation, a high-speed mechanical-mixing method was applied.

The physicochemical and thermochemical characterization of the prepared fuel blends was performed in the laboratories of the Department of Chemical Engineering at Atatürk University, and density, kinematic viscosity, calorific value, cloud point, and pour point were determined in this scope (Table 2).

**Table 2:** Equipment and Methods Used in Fuel Characterization

analysis	equipment	brand/model	standard method
density (15 °C)	automatic density meter	Rudolph Research Analytics, DDM 2909	TS EN ISO 12185
kinematic viscosity (40 °C)	digital constant temperature kinematic viscosity bath	Köhler KV4000 Series	TS EN 1451 ISO 3104
upper calorific value	bomb calorimeter	IKA C-200	ASTM D 240
cloud point	turbidity and pour point bath	SETA (11010-2)	TS EN 116
pour point	turbidity and pour point bath	SETA (11010-2)	TS 1233 ISO 3016
Fourier transform infrared (FT-IR) analysis	FT-IR spectrophotometers	VERTEX 70v usage	---

FT-IR (Fourier transform infrared) spectroscopy was used to identify the molecular structures, chemical bond characteristics, and functional groups of fuel samples, which ensures fuel quality, safety, and operational reliability of the fuel blends. The analyses were performed using a Bruker VERTEX 70v spectrophotometer in the 4000–400 cm^–1^ wavenumber range and at a spectral resolution of 4 cm^–1^. Characteristic spectral fingerprints were obtained for pure diesel (D100), and the prepared blended fuel samples (WTRO30, WTRO10WTPO20, WTRO20WTPO10, and WTPO30) were extracted; the presence of aliphatic and aromatic hydrocarbon components in the structure and functional groups determining the physicochemical properties of the fuel was qualitatively confirmed.

As presented in Table 3, distillation curves for pure diesel, WTPO, WTRO, and their blends were obtained and analyzed to evaluate critical parameters directly related to the thermodynamic behavior of the fuel, such as cold-start performance, combustion efficiency, and exhaust emissions.

**Table 3:** Distillation Characteristics of Test Fuels (ASTM D86)^a^

fuel sample	IBP (°C)	T10 (°C)	T50 (°C)	T90 (°C)	FBP (°C)
D100	168	198	268	338	358
WTRO30	172	206	276	346	362
WTRO10WTPO20	175	212	284	354	370
WTRO20WTPO10	170	204	272	342	360
WTPO30	182	224	298	368	382

a A brief discussion of the distillation characteristics and their implications for cold-start behavior and combustion has also been included in Subsection [Sec sec3.1].

### 2.2 Engine Tests and Emission Measurements

#### 2.2.1 Experimental Setup and Procedure

Engine performance and exhaust emission tests were conducted at an experimental engine test rig, detailed in Figure 1, located in the Engine Test Laboratory at Dicle University, which comprises a single-cylinder, four-stroke, air-cooled, CI engine.

**Figure 1:**
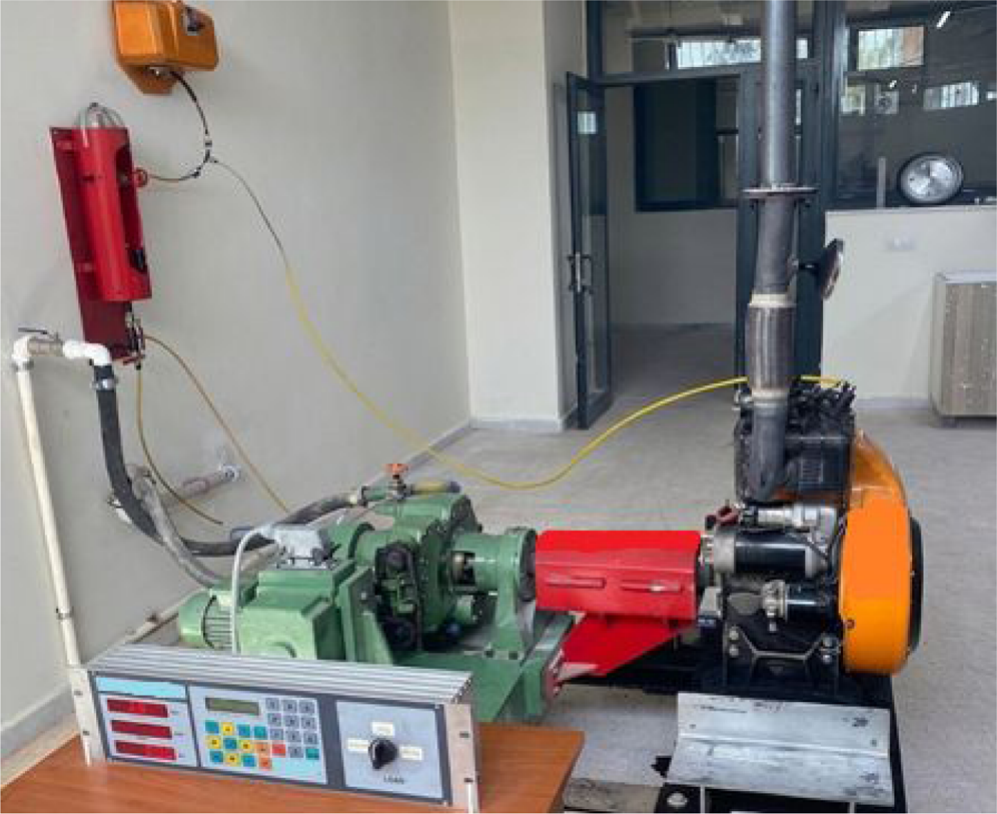
Physical engine test system in the laboratory.

As detailed in the schematic view given in Figure 2, the test rig features a water-braked dynamometer for controlling the engine load, an electronic control unit (ECU) for monitoring speed and torque data, a fuel consumption measurement system for accurate determination of fuel consumption, and an exhaust gas analyzer for determining exhaust emissions.

**Figure 2:**
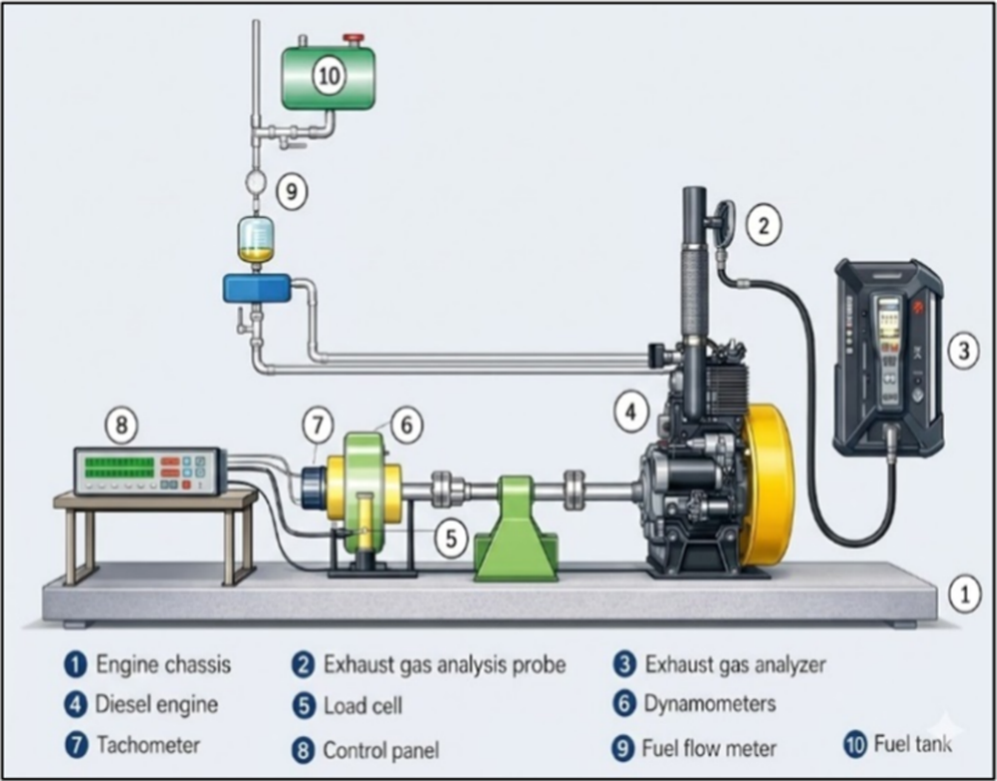
Schematic view of the engine test rig.

The technical specification of the diesel engine is presented in Table 4.

**Table 4:** Technical Specifications of the CI Test Engine

parameters	specification
engine models	Antor 4 LD 820
engine type	four-stroke, single cylinder
fuel type	diesel
issue	102 mm
paralysis	100 mm
swept volume	817 cm^3^
compression ratio	17:1
cooling type	air-cooled
maximum engine speed	3000 rpm
maximum power	12.7 kW at 3000 rpm
maximum torque	50 N m at 1600 rpm
injector nozzle opening pressure	20 MPa (mechanically controlled)
fuel injection timing	24° CA before top dead center (bTDC)
specific fuel consumption	255 g/kWh at 2800 rpm
engine position	vertical

The test procedure was initiated by running the engine at 3000 rpm for 10 min without a load to achieve thermal stability. When switching between different fuel samples, the fuel line and tank were completely drained to ensure that the system was free of previous fuel residues. After the fuel sample to be tested was fed into the system, the load was gradually applied to the engine through the dynamometer, and the engine speed was reduced to the respective engine speed. The engine was allowed to run for 5 min at each speed level to ensure stabilization. Torque, power, and SFC data were then recorded via an ECU screen.

The experimental procedure applied for each blend is presented in the flowchart given in Figure 3.

**Figure 3:**
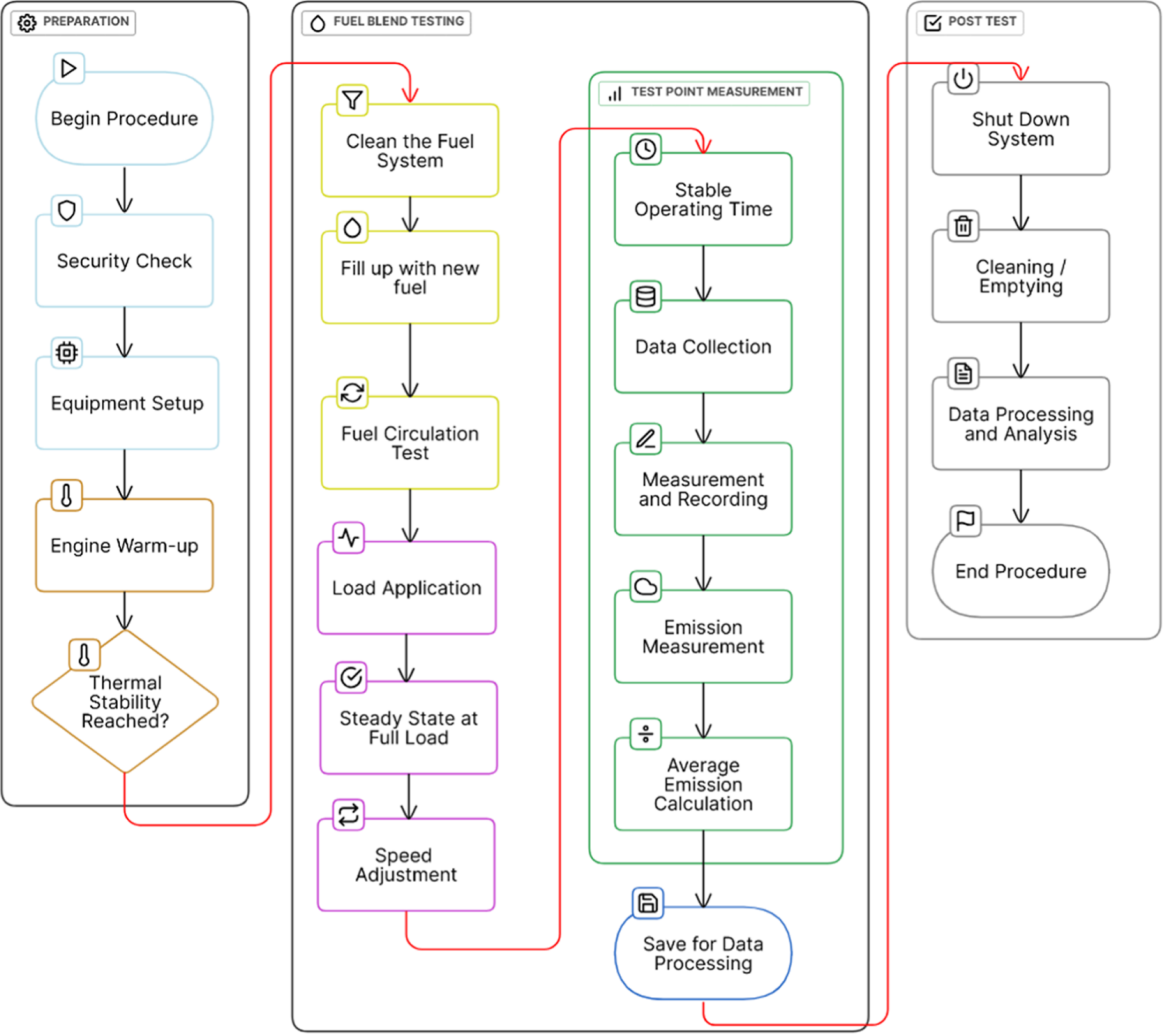
Flowchart for the test procedure.

The determination of CO, NO, and NO_*x*_ emissions was performed using a Testo 350 exhaust gas analyzer with high measurement accuracy, as presented in Table 5. To ensure the representativeness and stability of the gas sampling, the data were recorded by integrating the analyzer probe into the exhaust line through a special measurement port located 30 cm below the exhaust manifold. Emission data were sampled at 50 s intervals under steady-state conditions at each speed step to increase measurement accuracy. All performance and emission data were statistically organized and subjected to a graphical analysis.

**Table 5:** Technical Specifications of the Testo 350 Exhaust Gas Analyzer

parameters	measuring range	accuracy
CO (carbon monoxide)	0–10,000 ppm	±10% of reading
NO (nitrogen oxide)	0–4000 ppm	±10% of reading
NO_2_ (nitrogen dioxide)	0–500 ppm	±5% of reading
exhaust gas temperature	–200 to 1370 °C	±1 °C

Under each test condition, exhaust emission data were continuously recorded in 50 s intervals, and each measurement was performed in three independent repetitions. To minimize possible momentary fluctuations and variability in the measurements, the arithmetic mean of these three independent data points was defined as the characteristic emission value for the relevant operating condition. This statistical approach increased the reliability of the experimental data and consistency of the results. Yet, to ensure the statistical reliability of the results despite the limited number of repetitions, standard deviations were calculated for all measured parameters, and the data dispersion is visually represented via error bars on all of the corresponding charts.

#### 2.2.2 Calculation of Engine Performance Parameters

The basic parameters used in the calculations are engine speed (*n*) and torque (*T*), the rotational force (Nm) produced at the flywheel output of the engine. The product of these two variables constitutes the engine’s total brake power (BP):

1 $$BP=\frac{2\pi nT}{60}10^{-3}\;\left(kW\right)$$

In eq 1, *n* represents the engine speed in rpm and *T* represents the engine torque in Nm.

The mass of fuel consumed per unit of BP output, known as BSFC, is one of the most critical performance indicators that determine the fuel efficiency and combustion quality of an engine and is obtained by dividing the mass fuel flow rate by BP:

2 $$BSFC=\frac{{ṁ}_{f}}{BP}\;\left(g/kWh\right)$$

The expression given by eq 2 represents the fuel flow rate as ${ṁ}_{f}$ (kg/h) and the engine power as BP.

Volumetric fuel consumption was determined using a graduated buret having a capacity of 50 mL and a precision of 1 mL. The buret system was integrated in series between the fuel tank and the engine supply line. After the engine reached thermal stability at each test point, the time taken to consume 50 mL of fuel was recorded using a digital stopwatch with an accuracy of 0.01 s.

The fuel mass flow rate ( $\dot{m_{f}}$ ) was calculated based on the measured volumetric consumption in *t* time and the density of the fuel at that ambient temperature:

3 $${ṁ}_{f}=\frac{\rho_{f}\times V_{f}\times 3600}{t}\;\left(kg/h\right)$$

where ρ_f_ (kg/m^3^) is the density of the fuel, *V*_f_ (m^3^) is the volume of the fuel consumed by the engine over a time period *t* (s), and *t* is the duration required to consume the fuel volume *V*_f_.

Brake thermal efficiency (BTE) indicates how much of the chemical energy of the fuel (based on LHV) is converted into effective mechanical power. This parameter is widely used to compare the energy conversion efficiency of an engine in different fuel/mixture fuel applications

4 $$BTE=\frac{BP}{{ṁ}_{f}\cdot LHV}$$

## 3 Results and Discussion

### 3.1 Physicochemical Properties of the Prepared Blends

The physicochemical characteristics of the prepared fuel blends play a critical role in determining the combustion behavior and performance limits of these fuels in CI engines. The operational performance of fuels under cold climate conditions is directly related to their pour and cloud points.

Comprehensive characterization results of D100 and WTRO30, WTRO10WTPO20, WTRO20WTPO10, and WTPO30 blends were obtained by using advanced analytical techniques. These tests and analyses provided fundamental insight into the combustion behavior of the blends and were aimed to assess their viability as alternative drop-in fuels for short-term use without requiring engine modifications. Table 6 presents the thermophysical properties of the tested fuels against ASTM D975, EN 14214, and ASTM D6751 standard limits.

**Table 6:** Physicochemical Properties of Tested Blends^a^

fuel blend	density @15 °C (kg/m^3^)	viscosity @ 40 °C (mm^2^/s)	cloud point (°C)	pour point* (°C)	LHV (MJ/kg)
D100	830	3.00	+2	–28	41.922
WTRO30	850	4.30	–5	≤−40	42.235
WTRO10WTPO20	840	2.74	–23	≤−40	41.176
WTRO20WTPO10	850	3.16	–22	≤−40	41.468
WTPO30	860	2.84	–20	–28	40.897
ASTM D975 ^36^	820–860	2.0–4.5	–15 to −5	–35 to −15	-
EN14214 ^35,36^	860–900	3.5–5.0	-	-	-
ASTM D6751 ^36^	575–900	1.9–6.0	–3 to −12	–15 to −16	-

a According to the findings, the density values of all samples ranged between 830 and 860 kg/m^3^, while their kinematic viscosities varied between 2.74 and 4.3 mm^2^/s. Both density and viscosity data fully comply with the limits specified in the ASTM standards. The highest viscosity value (4.3 mm^2^/s) was recorded in the WTRO30 sample, which had the highest WTRO ratio. This can be explained by the initial high-viscosity characteristic of the waste transformer oil.^37^

Although EN14214 and ASTM D6751 are biodiesel standards, they are deliberately and inevitably referenced since there is currently no specific standard for blends of diesel fuel with waste-derived oils such as WTRO, and WTPO. On the other hand, these standards provide the means for the findings of this study be comparable with other alternative fuel studies in the literature.^11,12,33–36^

When examining the Table 6 data, it is observed that all blends containing WTRO have very low pour points. In contrast, pure diesel and blends containing 30% waste tire pyrolytic oil (WTPO30) have higher pour points;^38^ a waste transformer oil with a low pour point significantly improves the cold flow properties of the fuel when included in the blends. The pour points of all samples remained within standard limits. On the other hand, although no specific standard limit has been defined for pour point, cloud point, and calorific value, the obtained values are seen to be consistent with the literature and at acceptable levels. The lower calorific values (LHV) of the samples were quite close to each other and averaged 41 MJ/kg.

These tests and analyses provided fundamental insight into the combustion behavior of the blends and were aimed to assess their viability as alternative drop-in fuels for short-term use without requiring engine modifications. The analyses confirmed that all test fuels fully met the criteria set for alternative diesel fuels and that they could be used as drop-in alternatives. However, detailed analyses for the cetane number or derived cetane number, lubricity (HFRR), and sulfur content should be performed in order to comprehensively evaluate long-term engine durability.

### 3.2 Fourier Transform Infrared Spectroscopy (FT-IR)

The infrared spectra of D100, WTRO30, WTRO10WTPO20, WTRO20WTPO10, and WTPO30 and their superimposed spectra are shown in Figure 4.

**Figure 4:**
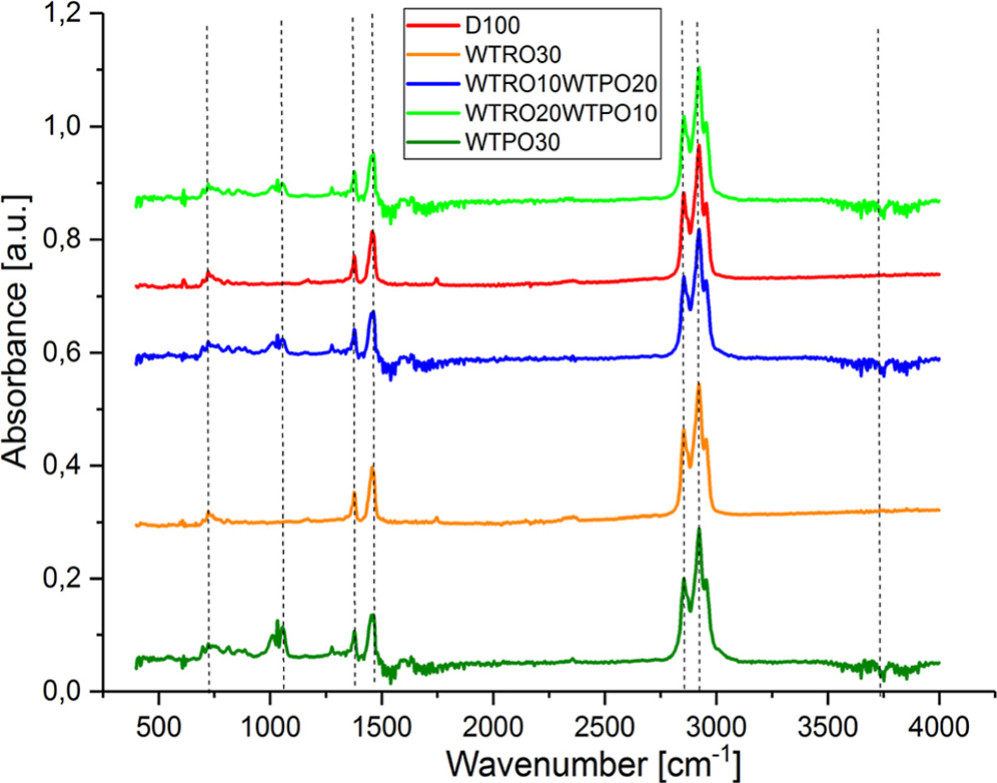
Infrared spectra for D100, WTRO30, WTRO10WTPO20, WTRO20WTPO10, and WTPO30.

Because of the petroleum-derived hydrocarbon nature of all samples, common characteristic peaks were observed in the spectra. The intense peaks observed in the 2950–2850 cm^–1^ range originate from the C–H stretching vibrations of the paraffinic and naphthenic components in the structure. In addition to the 1460–1450 cm^–1^ (CH_2_ bending) and 1375–1380 cm^–1^ (CH_3_ symmetric deformation) bands characterizing the aliphatic chain structure, the 720–730 cm^–1^ (CH_2_ rocking) peaks indicate the presence of long paraffinic chains. In the pure diesel (D100) sample, aliphatic hydrocarbons (alkanes and alkenes) were dominant, and the aromatic bands were found to be quite weak. In contrast, in the WTRO30 blend containing waste transformer oil, ester carbonyl (C=O) structures became prominent due to oxidative aging to which the transformer oil was exposed during its service life. In binary and ternary blends containing waste pyrolytic oil (WTPO30, WTRO10WTPO20, and WTRO20WTPO10), bands representing aromatic C=C and =C–H bonds showed a characteristic increase due to the intense aromatic components inherent in the pyrolysis process. The specific absorbance band ranges for the samples and the functional groups attributed to these bands are summarized in Table 7.^39,40^

**Table 7:** Absorption Band Ranges for Fuel Samples D100, WTRO30, WTRO10WTPO20, WTRO20WTPO10, and WTPO30

absorption bands (cm^–1^)	sample type	functional group
2950–2850	all fuel samples	aliphatic C–H stretching vibration (asymmetrical/symmetrical) aliphatic C–H stretching vibration (asymmetrical/symmetrical)
1760–1750	WTRO30	aster carbonyl C=O stretching vibration
1650–1600	WTRO10WTPO20	aromatic C=C stretching vibration
	WTRO20WTPO10	
	WTPO30	
1460–1450	all fuel samples	CH_2_ scissoring
1380–1375	all fuel samples	CH_3_ bending vibrations
1300–1100	WTRO10WTPO20	C–O stretching vibration (alcohols and esters)
	WTRO20WTPO10	
	WTPO30	
900–700	WTRO10WTPO20	aromatic C–H bending vibrations (out-of-plane bending)
	WTRO20WTPO10	
	WTPO30	
730–720	all fuel samples	CH_2_ rocking

The aliphatic groups found in pure diesel ensure a clean combustion and low soot formation. It has low lubrication properties and a short ignition delay. In blends containing transformer oil, in addition to the aliphatic groups found in diesel fuel, oxygenated compounds formed due to the aging of transformer oil facilitate fuel combustion. Transformer oil enhances the lubricating properties of fuel because it contains heavier, more-viscous compounds that interact with the surface. Blends containing waste tire pyrolytic oil include aliphatic, aromatic, and oxygenated compounds. Aromatic compounds present in the structure increase ignition delay, soot, CO, and NO_*x*_. Oxygenated compounds partially balance this and improve the lubrication properties of the fuel.

### 3.3 Engine Performance Analysis

#### 3.3.1 Torque (*T*)

The torque graph presented in Figure 5 shows the torque variation for pure diesel (D100) and ternary blends (WTRO30, WTRO10WTPO20, WTRO20WTPO10, and WTPO30) as a function of engine speed (1200–2400 rpm) under full load conditions. The general trend is that the torque decreases as the speed increases for all fuels; this is consistent with typical diesel engine behavior and can be explained by the decrease in volumetric efficiency and the increase in friction losses.^41,42^

**Figure 5:**
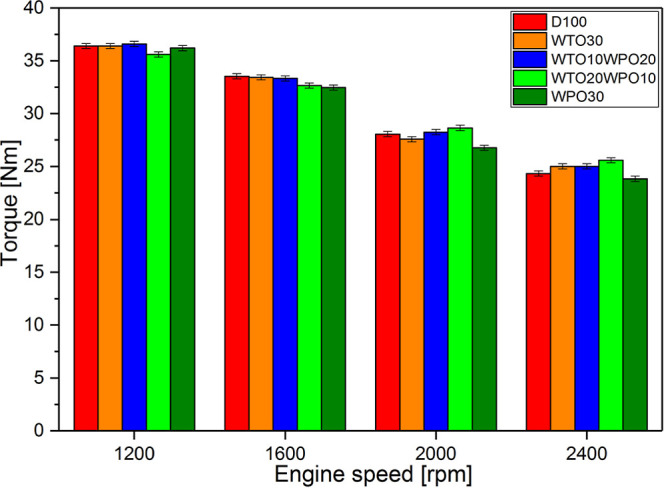
Change in brake torque (*T*) as a function of engine speed under full load conditions for blends D100, WTRO30, WTRO10WTPO20, WTRO20WTPO10, and WTPO30.

At low speeds (1200–1600 rpm), blends, especially WTPO30, produced lower torque than pure diesel, which can be attributed to WTPO’s low calorific value, high viscosity, and delayed combustion due to poor atomization. The literature also reports that high WTPO ratios lead to torque loss.^22,23,26^ Nevertheless, it is noteworthy that the WTRO20WTPO10 blend exhibited approximately a 3.66% increase in torque at high speeds (2000–2400 rpm), possibly due to the improved vaporization kinetics and combustion characteristics of the balanced effects of the ternary blend. It is noteworthy that the WTRO20WTPO10 blend exhibited approximately a 3.66% ± 0.42% increase in torque at high speeds (2000–2400 rpm). This improvement is attributed to the blend’s favorable physicochemical properties: its kinematic viscosity (3.16 mm^2^/s) and density (850 kg/m^3^) are very close to those of diesel fuel, promoting better atomization, faster evaporation, and more complete combustion. Additionally, the ternary combination balances the low-temperature flexibility of WTRO with the energy content of WTPO, while mitigating the negative effects of WTPO’s high aromatic content through dilution with diesel and WTRO.

When examining the average values, the most significant torque loss (2.49% decrease) was observed in WTPO30, while the WTRO10WTPO20 blend showed the best performance with a slight increase (0.72%). These findings support the torque improvements observed in separate WTRO studies^3,12^ and the acceptable torque levels at low ratios in WTPO binary blends,^17,18^ revealing that ternary blends at optimized ratios can provide torque levels close to or superior to pure diesel. In conclusion, high WTPO ratios cause performance loss, while balanced WTRO–WTPO combinations highlight the potential for sustainable alternative fuels.

It should be noted that the absolute torque values obtained in this study are lower than the manufacturer’s declared maximum torque (50 N m at 1600 rpm) due to the engine’s cumulative operating hours, component wear, and the use of alternative fuel blends with lower energy content and different combustion characteristics. However, the primary focus of this study is the comparative evaluation of the blends against diesel fuel under identical test conditions, for which the relative trends remain reliable.

#### 3.3.2 Brake Power (BP)

Figure 6 shows the changes in braking power with engine speed for the D100, WTRO30, WTRO10WTPO20, WTRO20WTPO10, and WTPO30 fuel blends in the 1200–2400 rpm engine speed range. At low speeds (1200–1600 rpm), waste oil blends (especially WTPO30 with a high WTPO ratio) produce lower braking power than pure diesel, which is due to the poor vaporization and combustion inefficiency associated with WTPO’s low calorific value and high viscosity. Similarly, the literature reports that binary blends with high WTPO content cause power loss at low rpmss.^22,23,26^ Yet, at 2000 and 2400 rpm values, the WTRO20WTPO10 blend induced an average power increase of 5.18%, which suggests that balanced effects of the ternary blend provide an advantage at high speeds due to better atomization and combustion kinetics.

**Figure 6:**
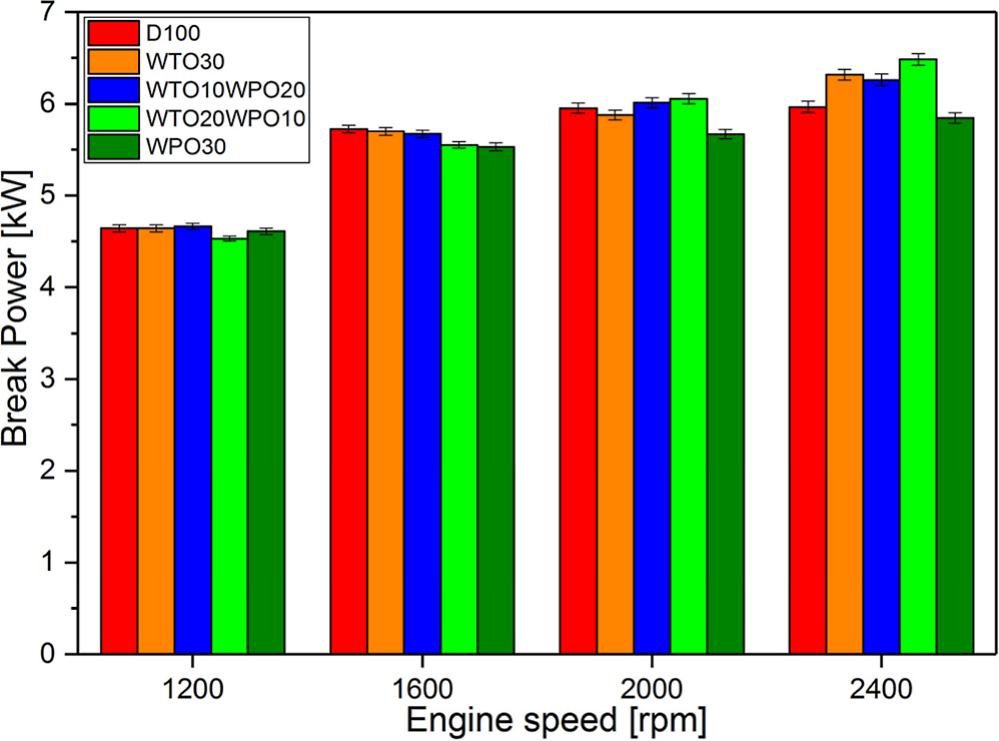
Change in braking power (BP) as a function of engine speed under full load conditions for blends D100, WTRO30, WTRO10WTPO20, WTRO20WTPO10, and WTPO30.

When average values are evaluated, the most significant power loss (2.84% decrease) was observed in WTPO30, while the WTRO20WTPO10 blend produced the highest braking power with a 1.47% increase. These results are consistent with the findings that report power improvements observed in separate WTRO studies^3,12,18^ and acceptable power levels in low-ratio WTPO blends.^17^ In conclusion, while high WTPO ratios cause overall power loss, balanced WTRO–WTPO combinations hold promising potential for sustainable fuels, as they can offer performance superior to or close to that of pure diesel, particularly at high speeds.

#### 3.3.3 Brake-Specific Fuel Consumption (BSFC)

The BSFC graph presented in Figure 7 shows the BSFC change for pure diesel (D100) and ternary blends (WTRO30, WTRO10WTPO20, WTRO20WTPO10, and WTPO30) as a function of engine speed (1200–2400 rpm) under full load conditions. The general trend is that BSFC increases as the engine speed increases for all fuels; this is a known behavior of diesel engines and can be explained by increased friction losses at high speeds, shorter combustion time, and a relative decrease in volumetric efficiency.^41,42^

**Figure 7:**
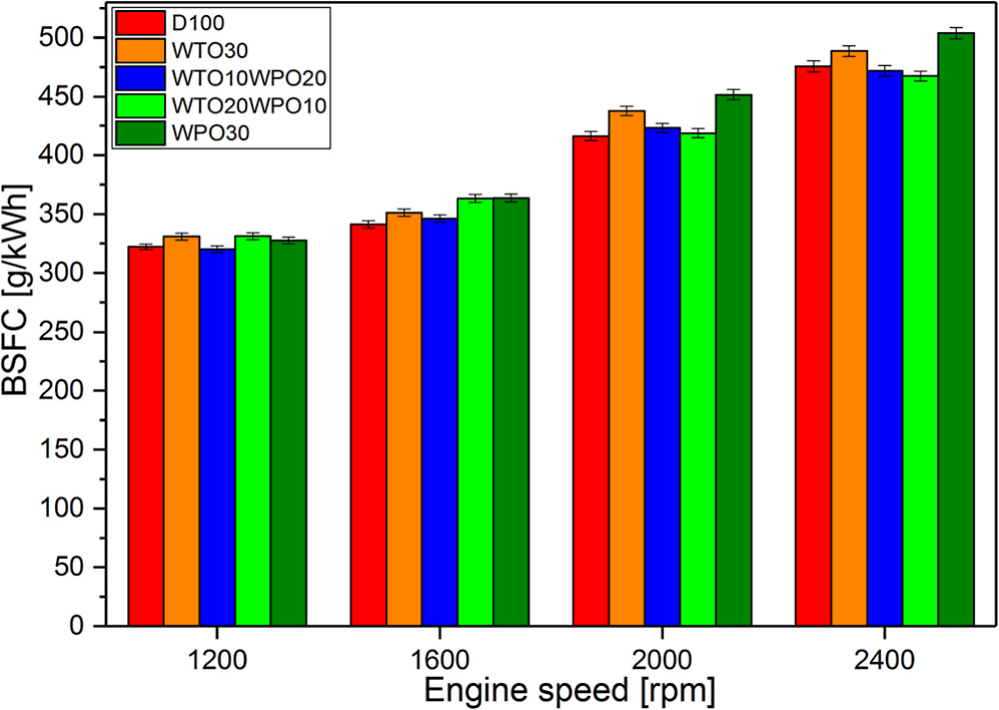
Change in BSFC as a function of engine speed under full load conditions for blends D100, WTRO30, WTRO10WTPO20, WTRO20WTPO10, and WTPO30.

Across the entire operating range, waste oil blends exhibit higher BSFC compared to pure diesel, with the most significant increase (5.90%) observed in the WTPO30 blend with a high WTPO ratio. This can be directly attributed to incomplete combustion and energy conversion inefficiency due to WTPO’s low lower heating value (LHV), high kinematic viscosity, and poor atomization characteristics—the literature frequently reports that binary blends with a similarly high WTPO content increase BSFC.^22–24,26^ However, the WTRO10WTPO20 blend provided fuel economy closest to pure diesel with only a minimal increase of 0.4%.

While separate WTRO studies have observed that low-ratio blends have a limited effect on BSFC or even reduce it,^3,12^ findings that high WTPO ratios negatively affect fuel consumption^17,29^ are consistent with the results of this study. Consequently, while optimized ratios among ternary blends (e.g., WTRO10WTPO20) offer acceptable fuel economy, blends with a high WTPO content create disadvantages in terms of energy efficiency.

#### 3.3.4 Brake Thermal Efficiency (BTE)

BTE indicates how much of the fuel’s chemical energy a motor converts into useful mechanical work at the crankshaft. It is calculated as the amount of fuel consumed per unit of brake power output and expressed as a percentage. Higher BTE values indicate better energy conversion efficiency performance and more effective use of fuel energy.

Figure 8 shows the change in BTE with engine speed for all blends (D100, WTRO30, WTRO10WTPO20, WTRO20WTPO10, and WTPO30) tested under full-load conditions. The general trend is for BTE to decrease slightly or plateau as engine speed increases; this is consistent with the limitation of energy conversion efficiency in diesel engines due to increased friction losses and reduced combustion duration at high speeds.^41,42^

**Figure 8:**
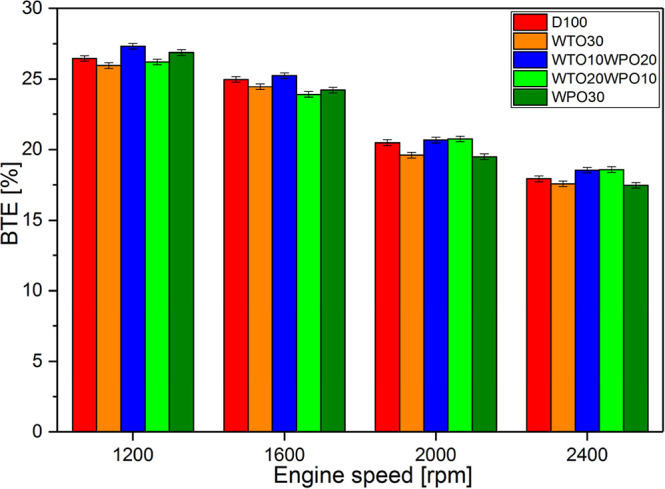
Change in brake BTE as a function of engine speed under full load conditions for blends D100, WTRO30, WTRO10WTPO20, WTRO20WTPO10, and WTPO30.

Experimental results show that all waste-derived blends achieved higher BTE values compared to the base D100 diesel fuel across the entire tested speed range (1200–2400 rpm). When examining Figure 8, the average brake BTE values of all fuel samples containing waste transformer oil and waste tire pyrolytic oil additives at all engine speeds, when compared to the D100 fuel sample, the highest BTE value was observed in the WTRO10WTPO20 fuel sample with a 2.14% increase, while the lowest BTE value was observed in the WTPO30 fuel sample with an 11.66% decrease.

Throughout the entire temperature range, the BTE values of ternary blends are close to or occasionally superior to pure diesel, with the most notable decrease (11.66% reduction) observed in the WTPO30 blend due to incomplete combustion caused by WTPO’s low calorific value, high aromatic content, and poor vaporization associated with its viscosity. Although, high WTPO content significantly reduces BTE,^22–24,26^ the WTRO10WTPO20 ternary blend is quite promising, exhibiting the highest BTE with a 2.14% increase over D100.

Reports that low-ratio blends preserve or increase BTE^3,4,12^ and that acceptable efficiency levels are achieved at low WTPO ratios^17,29^ are consistent with these results. In conclusion, the WTRO10WTPO20 ternary blend could offer superior performance to pure diesel and gain practical applicability of waste-derived sustainable fuels.

### 3.4 Exhaust Emission Analyses

All exhaust emission results presented in this section are reported as raw concentration values (ppm) measured directly by the exhaust gas analyzer. These values represent the volumetric concentration of each pollutant in the exhaust stream and are not converted to mass-based emission rates (e.g., g/kWh) as the primary focus of this study is the comparative evaluation of fuel blends under identical engine operating conditions.

#### 3.4.1 Carbon Monoxide (CO) Concentrations

The carbon monoxide (CO) emission graph presented in Figure 9 shows the CO emission changes for pure diesel (D100) and ternary blends (WTRO30, WTRO10WTPO20, WTRO20WTPO10, and WTPO30) as a function of engine speed (1200–2400 rpm) under full load conditions. The general trend is that CO emissions show a slight decrease or remain stable as engine speed increases; this is consistent with increased in-cylinder turbulence at high speeds and the partial combustion reduction due to air blend homogeneity.^41,42^

**Figure 9:**
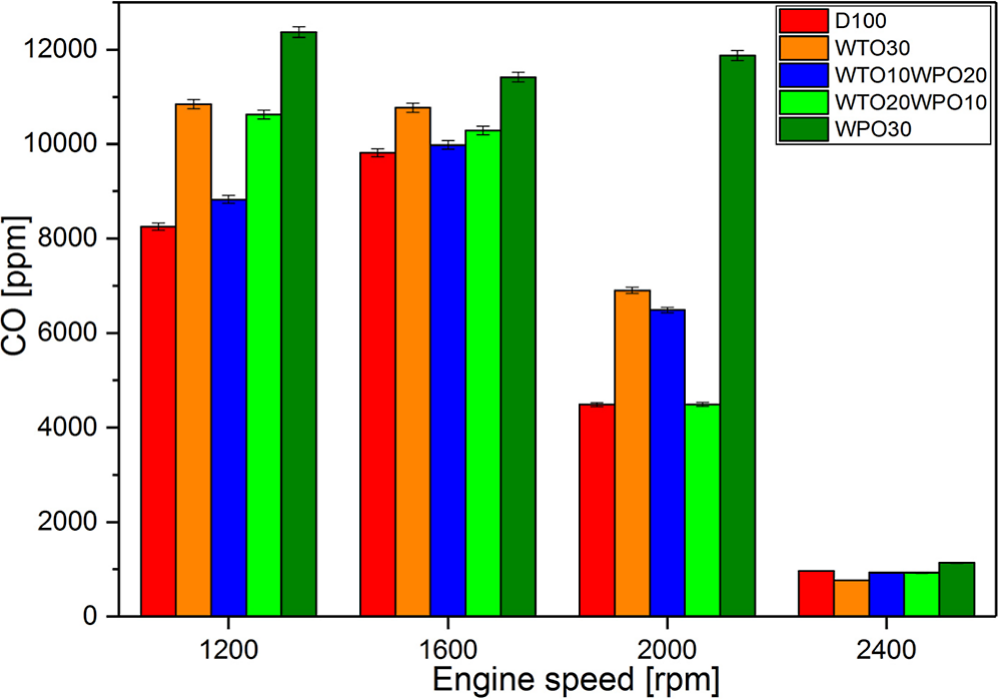
CO variation with engine speed under full load conditions for D100, WTRO30, WTRO10WTPO20, WTRO20WTPO10, and WTPO30 fuel blends.

Ternary blends produce significantly higher CO emissions than pure diesel across the entire operating range, with the most dramatic increase (56.30%) recorded in the WTPO30 blend with a high WTPO content. FT-IR analysis had shown the presence of aromatic compounds in blends containing waste tire pyrolytic oil. As the proportion of waste tire pyrolytic oil in the fuel blend increases, the proportion of aromatic components will also increase. Due to their high stability, aromatics are difficult to oxidize completely. This increases CO formation during combustion. Therefore, this significant increase in the WTPO30 blend is directly attributed to incomplete combustion resulting from poor atomization and vaporization characteristics due to WTPO’s high aromatic hydrocarbon content. Similarly, the literature frequently reports that binary blends containing high WTPO significantly increase CO emissions.^22–24,26^ In contrast, the WTRO10WTPO20 blend at balanced ternary ratios exhibited the lowest increase (11.58%) and the CO profile closest to pure diesel; this indicates that appropriate WTRO addition can improve combustion quality by partially balancing viscosity.

Findings from separate WTRO studies indicating that low-ratio blends have a limited effect on or reduce CO emissions^3,4,12^ and that acceptable CO levels are achieved at low WTPO ratios^17,29^ are consistent with these results. This significant increase is primarily attributed to incomplete combustion resulting from poor atomization and vaporization characteristics, which are associated with WTPO’s high kinematic viscosity (2.84 mm^2^/s) and low cetane number. Additionally, the FT-IR spectra of WTPO30 showed increased intensity in the aromatic C=C and =C–H absorption bands (1650–1600 cm^–1^ and 900–700 cm^–1^), suggesting a higher proportion of aromatic compounds compared to diesel. Aromatic hydrocarbons are known to have higher C/H ratios and greater resistance to thermal decomposition, which may contribute to incomplete combustion and higher CO formation.^41,43^ However, without quantitative analysis, the exact contribution of aromatics cannot be isolated from other fuel properties.

#### 3.4.2 Nitrogen Oxide (NO) Concentrations

Figure 10 shows the variation in NO emissions with engine speed for all blends (D100, WTRO30, WTRO10WTPO20, WTRO20WTPO10, and WTPO30) tested under full-load conditions. The general trend is that NO emissions decrease as the engine speed increases for all fuels; this can be explained by longer combustion times at low speeds and high cylinder temperatures promoting thermal NO formation via the Zeldovich mechanism.^41,42^

**Figure 10:**
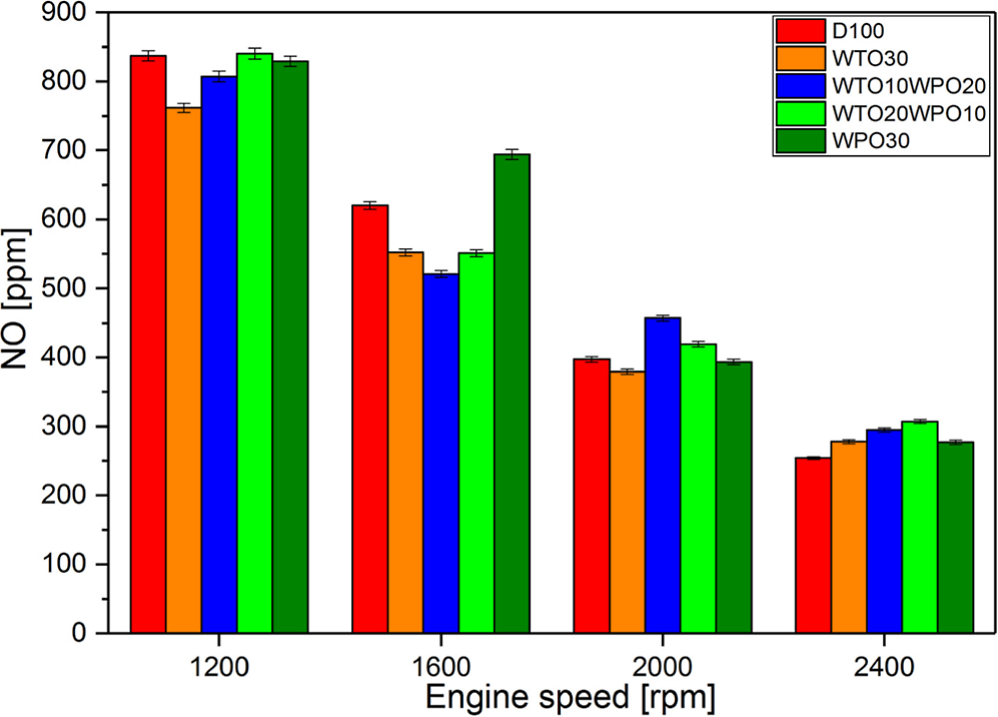
Variation of NO emissions for all fuel types across different engine speeds.

When examining Figure 10, the average NO emission values at all engine speeds for all fuel samples containing waste transformer oil and waste tire pyrolytic oil additives, compared to the D100 fuel sample, show that the highest NO emission value was observed in the WTPO30 fuel sample with a 4.03% increase, while the lowest NO emission value was observed in the WTRO30 fuel sample with a 6.50% decrease.

NO emissions from ternary blends remain close to or slightly lower than pure diesel across the entire operating range, with the most significant increase (4.03%) observed in the WTPO30 blend with a high WTPO content. This slight increase is attributed to the formation of local high-temperature zones due to the relatively high aromatic content of WTPO30, which contains the highest amount of waste tire pyrolytic oil, and its differing combustion characteristics. The literature frequently reports that binary blends containing high WTPO increase NO emissions.^22–24,26^ In contrast, the WTRO30 blend exhibited the lowest NO emissions, showing a 6.50% reduction compared to pure diesel; this positive effect is thought to stem from WTRO’s lower oxygen content and its ability to limit combustion temperatures.

Findings from separate WTRO studies showing that low-ratio blends reduce NO/NO_*x*_ emissions^3,11,12^ and that slight increases in NO are observed at low WTPO ratios^17,29^ are highly consistent with these results. Consequently, ternary blends with high WTPO content cause a limited increase in NO formation, while WTRO-dominant blends (e.g., WTRO30) provide significant NO_*x*_ reduction, offering environmental advantages; this highlights the critical role of ratio selection in emission optimization for waste-oil-based alternative fuels.

#### 3.4.3 Total Nitrogen Oxide (NO_*x*_) Concentrations

The total nitrogen oxide (NO_*x*_) emission graph presented in Figure 11 shows the NO_*x*_ emission variation for pure diesel (D100) and ternary blends (WTRO30, WTRO10WTPO20, WTRO20WTPO10, and WTPO30) as a function of engine speed (1200–2400 rpm) under full load conditions. The general trend is that NO_*x*_ emissions decrease as the engine speed increases for all fuels; this behavior is explained by the fact that longer residence times and higher peak cylinder temperatures at low speeds increase thermal NO_*x*_ formation (Zeldovich mechanism), while at high speeds, the shorter combustion time and lower maximum temperatures reduce NO_*x*_ formation.^41,42^

**Figure 11:**
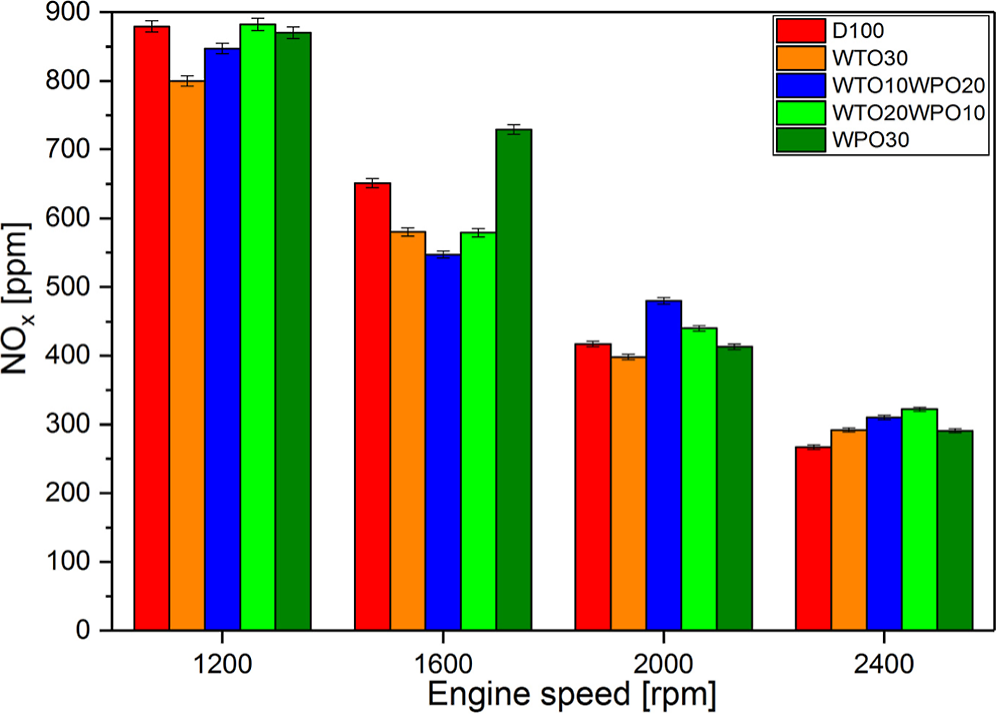
Variation of NO_*x*_ emissions for all fuel types across different engine speeds.

NO_*x*_ emissions from ternary blends remained close to or slightly below those of pure diesel across the entire operating range, with the most significant increase (4.02%) recorded in the WTPO30 blend with a high WTPO content. The increase being limited can be attributed to the high aromatic content of WTPO30 and the formation of local high-temperature zones due to the rapid combustion phase. Binary blends with high WTPO content were frequently reported to induce a slight increase in the NO_*x*_ emissions.^22–24,26^ The WTRO30 blend, however, produced the lowest NO_*x*_ emissions, with a 6.50% reduction compared to pure diesel owing to lower oxygen content, relatively low combustion temperature, and different thermochemical properties. The results obtained in this study are highly consistent with similar findings reported in the literature that states that low and medium WTPO blends significantly reduce NO_*x*_ emissions^3,4,11,12^ versus slightly higher NO_*x*_ emissions observed at low WTPO ratios.^17,29^ As a consequence, ternary blends with high WTPO content induce a limited increase in NO_*x*_, whereas blends with significant WTRO content (e.g., WTRO30) provide a remarkable reduction in NO_*x*_.

### 3.5 Uncertainty Analysis

To ensure the reliability of the experimental findings, an uncertainty analysis was performed using the square root sum method described by Kline and McClintock. The overall uncertainty of the calculated performance parameters (such as BTE and BSFC) depends on the individual accuracies of the independent measuring instruments (load cell, tachometer, buret, and stopwatch). The general equation for the relative uncertainty R of a calculated result, which is a function of the independent variables *x*_1_, *x*_2_, ..., *x*_*n*_, is given as follows:^43^

5 $$\frac{U_{R}}{R}=\sqrt{{\left(\frac{u_{1}}{x_{1}}\right)}^{2}+{\left(\frac{u_{2}}{x_{2}}\right)}^{2}+\cdot \cdot \cdot +{\left(\frac{u_{n}}{x_{n}}\right)}^{2}}$$

where *U*_R_ represents the overall uncertainty of the result and *u*_*i*_ represents the individual uncertainty of the measured independent variables. Overall uncertainties for the basic parameters were calculated based on the manufacturer’s specifications for the test setup components and the emission analyzer.

Uncertainty calculations for major performance indicator are presented below. As BP is a function of *T* and *N* as given in eq 1, the uncertainty in BP (δBP) is propagated from the uncertainties in *T* (δ*T*) and *N* (δ*N*) and is calculated as

6 $$\frac{\delta BP}{BP}=\sqrt{{\left(\frac{\delta T}{T}\right)}^{2}+{\left(\frac{\delta N}{N}\right)}^{2}}$$

The uncertainty in BSFC is a function of ${ṁ}_{f}$ and BP, as given in eq 2, and the propagation of these uncertainties is calculated as

7 $$\frac{\delta BSFC}{BSFC}=\sqrt{{\left(\frac{\dot{{\delta m}_{f}}}{\dot{m_{f}}}\right)}^{2}+{\left(\frac{\delta BP}{BP}\right)}^{2}}$$

Similarly, the uncertainty in BTE depends on the measurements of BSFC and LHV. The combined uncertainty is determined using

8 $$\frac{\delta BTE}{BTE}=\sqrt{{\left(\frac{\delta BSFC}{BSFC}\right)}^{2}+{\left(\frac{\delta LHV}{LHV}\right)}^{2}}$$

The overall uncertainties for all measured and calculated parameters are summarized in Table 8.

**Table 8:** Measurement Uncertainties for Experimental Parameters^a^

parameters	device/method	uncertainty
engine speed	tachometer	±%1.0
load/torque	load cell	±%1.0
fuel flow time	stopwatch	±%1.5
fuel mass	Buret	±%1.0
CO/NO, NO_*x*_	Testo 350 Analyzer	±%10/%5.0
brake power (BP)	BP = *f*(torque, speed)	1.41%
BSFC	BSFC = *f*(mass, time, BP)	2.29%
BTE	BTE = *f*(BSFC, LHV)	2.50%

a It was determined that the total experimental uncertainty for the overall test setup is within strictly acceptable limits for internal combustion engine research.

## 4 Conclusions

The main findings of the present study that experimentally investigated the effects of ternary blends of WTRO and WTPO with diesel fuel on the performance and exhaust emission characteristics of a single-cylinder, air-cooled, direct-injection CI engine can be summarized as follows:

- The physicochemical characterization results show that all prepared blends meet the ASTM D6751 and EN 14214 standard limits for density, kinematic viscosity, pour point, and calorific value, which indicates their suitability for use in diesel engines without hardware modifications.
- FT-IR spectroscopic analyses confirmed that all ternary blends (WTRO30, WTRO10WTPO20, WTRO20WTPO10, and WTPO30) have a petroleum-derived hydrocarbon-based structure. Aliphatic and aromatic functional groups were clearly detected in the spectra; a significant increase in the intensity of aromatic bands was observed as the WTPO ratio increased. In contrast, the WTRO content resulted in ester carbonyl structures becoming more dominant.
- The WTPO30 blend showed the highest performance losses: torque decreased by 2.49%, brake power by 2.84%, BSFC increased by 5.90%, and BTE decreased by 11.66%, primarily due to WTPO’s lower calorific value and higher viscosity.
- In terms of engine performance parameters, the most significant losses across the entire RPM range were observed in the WTPO30 blend, which has the highest WTPO content. For this fuel, an average decrease of 2.49% in torque and 2.84% in braking power was recorded, while BSFC increased by 5.90% and BTE decreased by 11.66%. These performance losses are attributed to WTPO’s low lower calorific value and poor atomization properties.
- In contrast, it was determined that balanced three-component blends exhibited performance particularly close to or better than pure diesel fuel in some parameters. The WTRO10WTPO20 blend provided a 0.72% increase in average torque and a 2.14% improvement in BTE (brake energy efficiency). The WTRO20WTPO10 blend showed an increase in the braking power of up to 5.18% at high engine speeds. These results demonstrate that combining WTRO with low-ratio WTPO in a balanced manner creates a positive synergy in terms of the performance.
- When evaluated in terms of exhaust emissions, the highest CO increase (56.30%) was observed in the WTPO30 blend. This situation is explained by the high aromatic content resulting from the high proportion of waste tire pyrolysis oil and the resulting incomplete combustion. The CO increase was more limited in other blends; the WTRO10WTPO20 blend exhibited the lowest increase rate of 11.58%, offering the most balanced profile in terms of emissions.
- NO and NO_*x*_ emissions generally showed a trend close to that of pure diesel fuel. While a limited increase in NO and NO_*x*_ emissions (4.02–4.03%) was observed in the WTPO30 blend, the WTRO30 blend provided a significant environmental advantage by achieving a 6.50% reduction in both parameters.

These findings demonstrate that WTRO and WTPO can be used as partial substitutes for diesel fuels in appropriate ternary blends. However, it should be noted that the experiments were conducted on a single-cylinder, air-cooled stationary engine, which differs significantly from modern multicylinder, turbocharged, common-rail diesel engines used in road transport.

## Data Availability

The data supporting this study are available within the manuscript and its supporting files. Fuel properties are presented in tabular form and via FTIR analysis. Engine performance and exhaust emission results are presented graphically throughout the manuscript.

## Nomenclature

ASTM: American Society for Testing and Materials
*T*: torque (Nm)
BP: brake power (kW)
BSFC: brake specific fuel consumption (g/kWh)
BTE: brake thermal efficiency (%)
CI: compression-ignition
DSC: differential scanning calorimetry
EGR: exhaust gas recirculation
FT-IR: Fourier transform infrared spectroscopy
HC: hydrocarbon (ppm)
CO: carbon monoxide (ppm)
CO_2_: carbon dioxide (ppm)
NO_*x*_: nitrogen oxides (ppm)
PM: particulate material (mg/m^3^)
WTPO: waste tire pyrolytic oil
WTRO: waste transformer oil
HCC: hemispherical open combustion chambers
RCC: recirculating combustion chambers
UHC: unburned hydrocarbons
HVO: hydrogen-treated vegetable oil
POB: palm oil biodiesel
DEE: diethyl ether
CPO: crude pyrolytic oil
CNG: compressed natural gas
*n*: engine speed
rpm: revolutions per minute
HRR_max_: maximum heat release rate
CP_max_: maximum cylinder pressure
NVH: noise, vibration, harshness
SCR: selective catalytic reduction
TG: thermogravimeters
D100: 100% diesel
WTRO30: 30%v/v WTRO + 70%v/v diesel
WTRO10WTPO20: 10%v/v WTRO + 20%v/v WTPO + 70%v/v diesel (ternary blend)
WTRO20WTPO10: 20% v/v WTRO + 10% v/v WTPO + 70%v/v diesel (ternary blend)
WTPO30: 30% v/v WTPO + 70%v/v diesel
